# The influence of alkyl chains on the performance of DSCs employing iron(ii) N-heterocyclic carbene sensitizers†

**DOI:** 10.1039/d1dt03252f

**Published:** 2021-10-20

**Authors:** Mariia Becker, Vanessa Wyss, Catherine E. Housecroft, Edwin C. Constable

**Affiliations:** Department of Chemistry, University of Basel BPR 1096 Mattenstrasse 24a 4058 Basel Switzerland

## Abstract

The photovoltaic performances of DSCs employing two new iron(ii) N-heterocyclic carbene (NHC) sensitizers are presented. The presence of *n*-butyl side chains had a significant impact on DSC performace. The improvement in DSC performance up to 0.93–0.95% was observed for a new heteroleptic sensitizer bearing one carboxylic acid anchoring group. The photovoltaic performance was remarkably affected by sensitization time and by a presence/absence of coadsorbent on the semiconductor surface. The highest photoconversion efficiencies (PCE) were achieved for DSCs sensitized over 17.5 hours without addition of coadsorbents. However, for a shorter dipping time of 4 hours, the presence of chenodeoxycholic acid improved the PCE from 0.46% (no coadsorbents) to 0.74%, respectively. The performance of DSCs based on a new homoleptic complex bearing two *n*-butyl side chains and a carboxylic acid anchor on each NHC-ligand was improved from 0.05 to 0.29% *via* changes in dye-bath concentration and sensitization time. The changes in the dye load on the semiconductor surface depending on the sensitization conditions were confirmed using solid-state UV-Vis spectroscopy and thermogravimetric analysis. Electrochemical impedance spectroscopy was used to gain information about the processes occurring at the different interfaces in the DSCs. The impedance response was strongly affected by the immersion time of the photoanodes in the dye-bath solutions. In the case of the homoleptic iron(ii) complex, a Gerischer impedance was observed after 17.5 hours immersion. Shorter dipping times resulted in a decrease in the resistance in the system. For the heteroleptic complex, values of the chemical capacitance and electron lifetime were affected by the immersion time. However, the diffusion length was independent of sensitization conditions.

## Abstract 2

DSC photoconversion efficiencies of up to 0.93–0.95% have been achieved for a new heteroleptic iron(ii) N-heterocyclic carbene sensitizer employing *n*-butyl chains.

## Graphical abstract

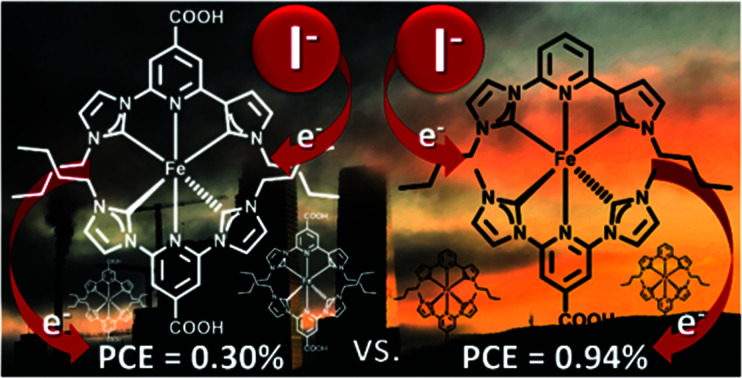

## Introduction

According to the Intergovernmental Panel on Climate Change (IPCC) that released its 6^th^ Assessment Report on the Physical Science Basis for climate change on August 2021,^1^ the man-made contribution to global warming resulted in numerous extreme incidents including tropical cyclones, heavy precipitation, heatwaves and droughts. Moreover, recent studies revealed that the rapid ice-mass loss is associated with horizontal motions of the Earth's crust.^2^ These fast changes are happening in the ocean, atmosphere and biosphere and will continue unless a reduction of greenhouse gases occurs.^3^ To cut carbon dioxide (CO_2_) emissions, fossil fuels have to be replaced with carbon neutral and green energy sources. Solar-to-electrical energy conversion has a crucial role in sustainable energy production. Dye-sensitized solar cells (DSCs) offer a low-cost alternative to well-known photovoltaics based on crystalline silicon. A typical *n*-type DSC consists of two electrodes, coated with conducting layers. A key part of a DSC is the photoanode (working electrode) which comprises an *n*-type semiconductor (commonly TiO_2_) deposited on a substrate (usually FTO-coated glass).^4^ A photosensitizer is adsorbed on the surface and provides an electron injection into the conduction band (CB) of a semiconductor under irradiation. The dye (or sensitizer) is chosen to provide a broad absorption in the visible region of the solar spectrum and to have a long-lived metal-to-ligand charge transfer (MLCT) excited state.^5^ Commonly used Ru(ii) polypyridyl complexes fulfil these requirements and offer photoconversion efficiencies (PCE) up to ≈12%.^5^ However, ruthenium suffers from being scarce and, therefore, expensive. Hence, Earth abundant metal complexes have become of great significance for DSC applications.

**Fig. 1:**
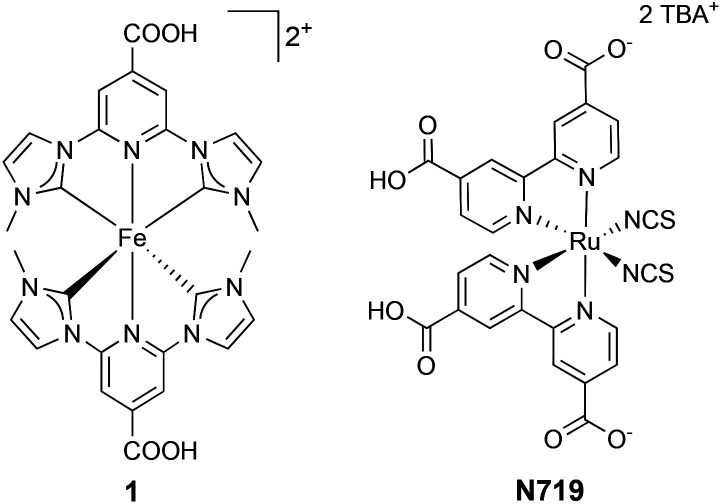
Structure of Fe(ii) NHC complex 1 and Ru(ii) polypyridyl complex N719.

Despite the low performance of first bis(2,2′-bipyridine)iron(ii) derivative as a sensitizer in DSCs,^6^ it was shown that iron-based dyes could, in principle, be used for photovoltaic applications. The limiting feature of iron(ii) polypyridyl complexes lies in their inefficient electron injection into the CB of the semiconductor. This is the result of fast deactivation from an MLCT to metal-centred (MC) state.^7^ In 2013, the first iron(ii) N-heterocyclic carbene (NHC) complex with an extended ^3^MLCT state lifetime of 9 ps was published by Wärnmark and co-workers, and DSCs sensitized with complex **1** (Fig. 1) gave 0.13% PCE.^8^ This breakthrough resulted in optimization studies of DSCs sensitized with **1**. In 2018, it was shown that the PCE could be improved from 0.13 to 0.57% (9.3% relative to N719 set at 100%) by optimization of electrolyte composition.^9^ Further electrolyte optimizations^10,11^ were done in combination with the application of the blocking underlayer on the photoanodes, which resulted in a PCE of near 1%.^12^ Gros and co-workers published a series of heteroleptic iron(ii) NHC complexes bearing a carboxylic acid functionality on one ligand.^13,14^ The push–pull strategy proved to be beneficial for a heteroleptic analogue of **1** with a PCE up to 1.27%. However, the introduction of phenyl and thienyl spacers to the anchoring ligand decreased the DSCs performance to 0.81 and 0.95%, respectively, in comparison to the analogous dye without spacers. It is important to note that the investigations were performed in the presence of a blocking layer under the semiconductor and in the presence of Mg^2+^ ions and tetrabutylammonium iodide in the electrolyte.^12^ Moreover, it has been shown that an iron(ii) NHC complex featuring a (porphyrinato)zinc(ii) conjugate stabilizes the ^3^MLCT state and increases its lifetime to 160 ps.^15^

One of the challenging aspects of dye **1** is rapid recombination of electrons injected into the conduction band (*e*_inj_) with the oxidized dye (sometimes referred to as a dye-cation).^16^ For the efficient DSC performance, the electron transfer from the CB to the oxidized dye has to be slower than the dye regeneration with the redox couple.^17^ However, the recombination of *e*_inj_ with the dye-cation may be supressed in the presence of chenodeoxycholic acid (CA) as has been shown by Durrant and co-workers with a Ru(ii)–phthalocyanine complex.^18^ On the other hand, alkyl chains can also offer a surface protection from this type of recombination.^19^ Using [Ru(dcbpy)(4,4′-dialkyl-2,2′-bipyridine)(NCS)_2_] (dcbpy = [2,2′-bipyridine]-4,4′-dicarboxylic acid) it was shown that the length of the alkyl substituents on the bipyridine moiety had an influence on the emission decay dynamics and, thus, on electron injection.^20^ Moreover, the observed recombination between injected electrons and the iodide/triiodide (I^−^/I_3_^−^) redox couple was less when the ancillary ligand contained an increased length of alkyl chains. The modification of N719 (Fig. 1) dye by attachment of nonyl substituents significantly decreased the recombination between *e*_inj_ and Co_2_^+/3+^ redox couples.^21^

The sensitization of the semiconductor surface with a dye is a subtle process which is affected by the nature of the dye molecule, solvent of electrode-immersion solution and sensitization time. If the dye molecules are too close to one another, dye⋯dye interactions may occur that could affect their charge characteristics.^22^ Dye aggregation is the result of intermolecular interactions including π-stacking of arene rings. This limitation can be overcome by the addition of coadsorbents^23,24^ such as chenodeoxycholic acid or the implementation of alkyl spacers in the dye structure.^25–27^ Furthermore, an excess of dye molecules in the dye-bath solution may result in the formation of multilayers on the TiO_2_ surface.^22^ In this case, the dye bath concentration together with the immersion duration have to be optimized.

**Fig. 2:**
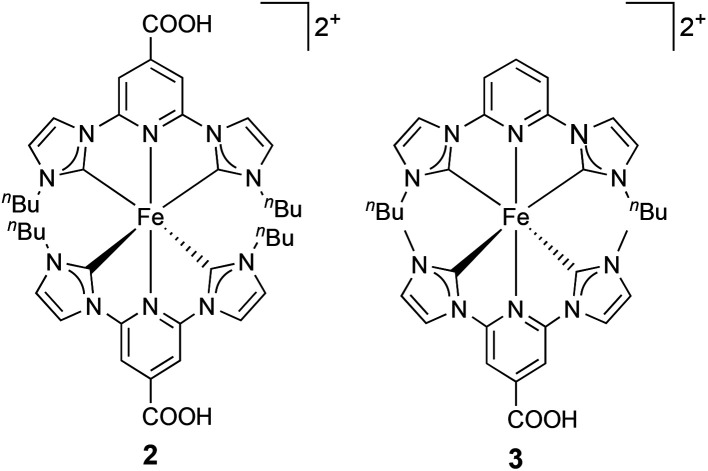
Structures of the sensitizers 2 and 3. Both complexes were isolated and used in DSCs as the [PF_6_]^−^ salts.

In this investigation, we present a study of DSCs based on new homoleptic and heteroleptic iron(ii) NHC complexes **2** and **3**, respectively (Fig. 2), with *n*-butyl functionalities, and investigate the optimization of the electrode sensitization process in terms of time, presence of coadsorbent and concentration of the dye-bath solution.

## Results and discussion

### Materials and methods

#### DSC fabrication

Commercial glass electrodes covered with FTO and screen-printed with TiO_2_ (Solaronix Test Cell Titania Electrodes, Solaronix SA, Aubonne, Switzerland) were rinsed with water, EtOH and dried on a heating plate at 450 °C for 30 min. Afterwards, the electrodes were cooled down to 60 °C and immersed in the dye bath. For Fe(ii) NHC complexes, the solvent was always MeCN. For the reference dye N719 (Solaronix SA, Aubonne, Switzerland), the dye-bath was a solution of N719 (0.30 mM) in EtOH and the dipping time was 17.5 hours. After sensitization, electrodes were rinsed with the solvent used in the dye bath and dried with a flow of nitrogen. Counter electrodes (Solaronix Test Cell Platinum Electrodes, Solaronix SA, Aubonne, Switzerland) were rinsed with water, EtOH and then heated at 450 °C for 30 min Afterwards, the electrodes were cooled down to 60 °C. The working and counter electrodes were combined with a thermoplast hot-melt sealing foil (Solaronix Test Cell Gaskets, 60 μm, Solaronix SA, Aubonne, Switzerland) by pressing them together while heating. Subsequently, the electrolyte was introduced into the space between the electrodes through a pre-drilled hole in the counter electrode by vacuum backfilling. The electrolyte composition for the Fe(ii) NHC complexes was LiI (0.18 M, I_2_ 0.05 M, 1-propyl-2,3-dimethylimidazolium iodide (0.60 M) in 3-methoxypropionitrile. The electrolyte composition for N719 was LiI (0.10 M, I_2_ 0.05 M, 1-butyl-3-methylimidazolium iodide (0.60 M), 1-methylbenzimidazole (0.50 M) in 3-methoxypropionitrile. The hole was sealed with hot-melt sealing foil and a cover glass (Solaronix Test Cell Sealings and Solaronix Test Cell Caps, Solaronix SA, Aubonne, Switzerland). At the end, silver paint (SPI Supplies, West Chester, PA 19381-0656, USA) was applied to the edges of each electrode from the FTO side.

#### Average values of photovoltaic parameters

According to statistical investigations based on DSCs sensitized with two different dyes (N719 and SQ2), the evaluation of data from multiple devices is required to validate DSC performance.^28^ Given a large enough data set, we have demonstrated that the use of average values is appropriate. Thus, in the following discussion, only average values for photovoltaic and EIS parameters will be used. Values for DSCs sensitized with N719 (used as a reference) are the average values based on four DSCs (Table S1†). Cell performances for multiple cells with the iron(ii) dyes were reproducible, and data for multiple devices are presented in the ESI (Tables S2–4†).

### Performance of DSCs

#### Photovoltaic performance

The photovoltaic performance was strongly affected by the time during which the working electrodes were immersed in solutions of the iron(ii) NHC sensitizers and the concentrations of the solutions. For the iron(ii) complex **1**, the typical dye bath conditions previously used in the literature were 0.50 mM in the presence of 0.10 mM of chenodeoxycholic acid in MeCN with immersion overnight.^9,16,29^ These conditions provided a starting point for the investigation of homoleptic complex **2**. The electrolyte composition was 0.18 M LiI, 0.05 M I_2_, 0.6 M 1-propyl-2,3-dimethylimidazolium iodide (PDMII) in 3-methoxypropionitrile.^10^

DSCs sensitized with **2** in the presence of the co-adsorbant CA exhibited a lower photovoltaic performance then those containing **1** with CA (Table 1). The low values of *J*_SC_, *V*_OC_ and ff resulted in a PCE of 0.05% (Table 1). Upon going from methyl to *n*-butyl side-chains (Fig. 1 and 2), steric effects could play an important role, and it was likely that the loading of the dye decreased on going from **1** to **2**. Thus, the CA was removed from the dye solution in an attempt to increase the dye loading sites on the semiconductor. However, the values of all parameters (*J*_SC_, *V*_OC_ and ff) decreased, leading to a lower PCE of 0.03% (Table 1). This effect could occur due to an increased rate of recombination between the semiconductor surface and redox-shuttle in the electrolyte that was suppressed in the presence of a CA. On the other hand, the increase in the dye load might lead to a higher rate of recombination between the dye molecules and the surface, or to quenching of excited states due to the dye aggregation on the surface.

**Table 1:** Average parameters for fully masked DSCs with dye **2** (0.50 mM). Data for multiple DSCs are shown in Table S2†

Dye^a^	Dipping time/h	*J*_SC_/mA cm^−2^	*V*_OC_/mV	ff/%	PCE/%	Rel. PCE^b^/%
Dye 1 CA	17.5	3.27	348	58	0.66	11.8^c^

Dye 2	17.5	0.58	114	46	0.03	0.5
Dye 2 CA	17.5	0.63	132	49	0.05	0.7
Dye 2 CA	4	1.31	256	64	0.22	3.4
Dye 2 CA	2	1.33	226	62	0.19	2.9

N719	17.5	14.70	651	66	6.36	100

a CA – in the presence of chenodeoxycholic acid (0.10 mM).

b Relative efficiencies are given with respect to N719 PCE set as 100%.

c Previously published data from ref. 10 (dye/CA 0.50 mM:0.10 mM).

In order to provide support for the latter, the immersion time of the electrodes in the dye solution was decreased from 17.5 to 4 hours in the presence of CA. The concentrations of the dye and CA were maintained (0.50 mM and 0.10 mM, respectively). The shorter sensitization time was beneficial for the performance of the DSCs. The values of *J*_SC_ and *V*_OC_ were approximately doubled compared to the 17.5 hours sensitization time, and the values of the fill factor increased from 49 to 64% (Table 1). The combined effect was an increase in PCE to 0.22%. A further decrease in the sensitization time to 2 hours had no significant impact on the photoconversion efficiencies (Table 1). However, it is noteworthy that the highest values of the fill factor and the open circuit voltage were observed for a dipping time of 4 hours. Thus, with a dye concentration of 0.50 mM and in the presence of 0.10 mM of chenodeoxycholic acid in MeCN, immersion times of 2 or 4 hours were beneficial to cell performance, and extended dipping times led to lower PCEs (Table 1).

**Fig. 3:**
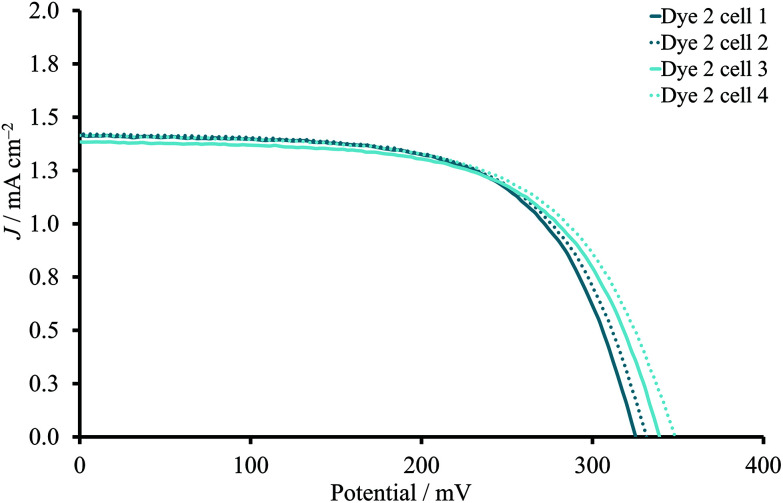
*J–V* curves to present the reproducibility for DSCs sensitized with **2** (0.05 mM) in the absence of CA. The dipping time was 2 hours.

Our unpublished optimizations of dye/coadsorbent concentration of complex **1**^30^ revealed that the best performing combination was dye **1** : CA in a 1 : 1 ratio with 0.05 mM concentrations of both dye and CA in MeCN due to the increased values of *J*_SC_ and *V*_OC_. The positive influence of shorter dipping times for **2** (Table 1) suggested the necessity of decreasing the dye bath concentration. Moreover, the reproducibility of these DSCs was not sufficient, indicating the necessity of further optimization. Therefore, the concentration of **2** was lowered from 0.50 mM to 0.05 mM, and corresponding DSCs were fabricated in the absence or presence of 0.05 mM CA after various immersion times (Table 2). Interestingly, the decrease in dye concentration in the sensitization solution had a beneficial impact on the PCE irrespective of the presence of CA in the mixture compared to 0.50 mM dye/0.10 mM CA. The *J*_SC_–*V*_OC_ trend could be described as an increase of short circuit current density and corresponding decrease of open circuit voltage on going from dye-bath times of 2 to 17.5 hours. Moreover, the changes in dye-bath concentration significantly improved the reproducibility of the DSCs (Fig. 3). For all DSCs, the PCE was in the range of 0.25–0.29% (average values, Tables 2 and S3†) for the different dipping times. The presence of CA had only a slight influence on the *V*_OC_ values, and the PCE was not significantly affected.

**Table 2:** Average parameters for fully masked DSCs with dye **2** (0.05 mM). Data for multiple DSCs are shown in Table S3†

Dye^a^	Dipping time/h	*J*_SC_/mA cm^−2^	*V*_OC_/mV	ff/%	PCE/%	Rel. PCE^b^/%
Dye 1	17.5	2.46	328	65	0.52	8.65^c^
Dye 1 CA	17.5	2.96	355	62	0.64	10.67^d^

Dye 2	17.5	1.64	270	63	0.28	4.4
Dye 2 CA	17.5	1.57	259	62	0.25	3.9
Dye 2	4	1.55	301	63	0.29	4.6
Dye 2 CA	4	1.56	277	63	0.28	4.4
Dye 2	2	1.41	336	63	0.29	4.6
Dye 2 CA	2	1.46	323	63	0.29	4.6

N719	17.5	14.70	651	66	6.36	100

a CA – in the presence of chenodeoxycholic acid (0.05 mM).

b Relative efficiencies are given with respect to N719 PCE set as 100%.

c Unpublished data from ref. 30 (dye 0.05 mM).

d Unpublished data from ref. 30 (dye/CA 0.05 mM:0.05 mM).

**Fig. 4:**
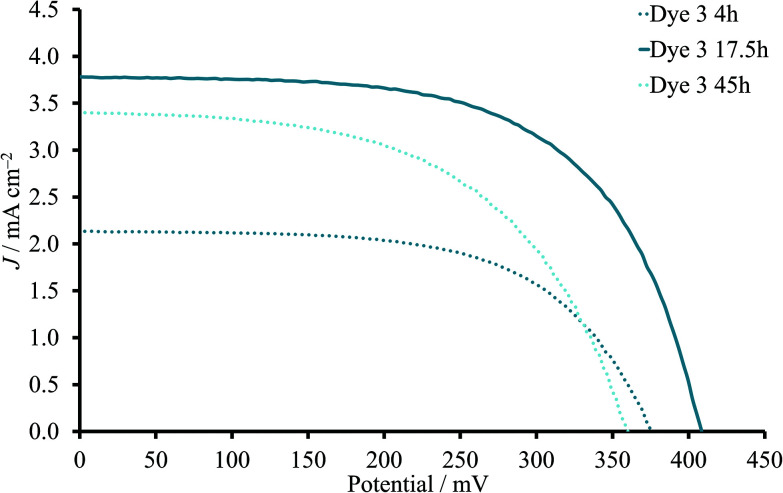
*J–V* curves for DSCs sensitized with **3** in the absence of CA. The dipping time was 4, 17.5 or 45 hours.

DSCs with the heteroleptic iron(ii) complex **3** (Fig. 2) were fabricated using a 0.05 mM dye-bath concentration. A PCE of 0.94% was achieved for a 17.5 hours sensitization time (Table 3). This approaches the record values for NHC-iron(ii) dyes reported by Gros and co-workers.^12^ A shorter dipping time of 4 hours resulted in significantly lower *J*_SC_ and *V*_OC_ values and a PCE of only 0.46% (Table 3). This trend was reproduced for a multiple cells (Table S4†). In contrast to the observations with homoleptic dye **2**, the addition of CA improved both *J*_SC_ and *V*_OC_ and, consequently, PCE values of DSCs with a 4 hours dipping time despite the slight decrease in ff (Table 3). At the same time, the presence of the CA in the DSCs with 17.5 hours sensitization decreased the PCE from 0.94 to 0.71% (Fig. 4). Thus, DSCs with CA had similar performances irrespective of the 4 or 17.5 hours dipping times (Tables 3 and S4†).

**Table 3:** Average parameters for fully masked DSCs with dye **3** (0.05 mM). Data for multiple DSCs are shown in Table S4†

Dye^a^	Dipping time/h	*J*_SC_/mA cm^−2^	*V*_OC_/mV	ff/%	PCE/%	Rel. PCE^b^/%
Dye 3	45	3.38	358	55	0.67	10.5
Dye 3	17.5	3.68	417	62	0.94	14.8
Dye 3 CA	17.5	3.00	397	60	0.71	11.2
Dye 3	4	2.05	370	61	0.46	7.3
Dye 3 CA	4	3.22	396	58	0.74	11.6

N719	17.5	14.70	651	66	6.36	100

a CA – in the presence of chenodeoxycholic acid (0.05 mM).

b Relative efficiencies are given with respect to N719 PCE set as 100%.

Since a longer sensitization time proved to be beneficial for dye **3** (17.5h compared to 4 h) in the absence of CA, the immersion was extended to 45 hours. However, the values of all three photovoltaic parameters decreased and led to lower PCE (average 0.67%, Table 3) compared to 17.5 hours.

#### UV-Vis analysis

**Fig. 5:**
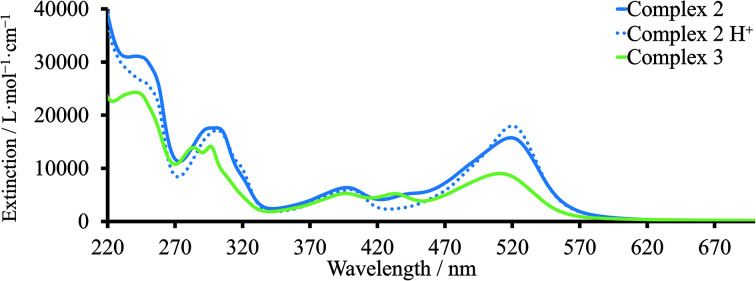
UV-Vis spectra of complexes **2** and **3** in MeCN.

The solution absorption spectra of dyes **2** and **3** are presented in Fig. 5. The spectrum of the homoleptic complex **2** exhibits two metal-to-ligand charge transfer (MLCT) bands at 397 and 521 nm, assigned to metal-to-carbene and metal-to-pyridine rings, respectively. These observations are in agreement with the UV-Vis spectrum for dye **1**.^8^ The absorption spectrum of dye **3** showed bands arising from transitions corresponding to MLCT from metal to the carbene centre at the same *λ*_max_ as **2** (≈396 nm). The MLCT band involving the pyridine ring functionalized with a carboxylic acid (Fig. 5) was blue-shifted to 510 nm in comparison to dye **2**. The third band corresponding to charge transfer to the non-substituted ligand was observed at 433 nm. The presence of this band was consistent with UV-Vis spectra previously published for related heteroleptic Fe(ii) NHC complexes.^29^ Interestingly, in the UV-Vis spectrum of **2** in MeCN, a shoulder at *λ*_max_ 430–450 nm was observed, which disappeared upon the addition of acid to the solution (Fig. 5). Moreover, this band was not observed in the solid-state UV-Vis spectra (discussed later). This effect was consistent with partial deprotonation of the carboxylic acid functionality in MeCN solution.

The solid-state UV-Vis (ssUV-Vis) spectra provide information about a dye adsorbed on the semiconductor. Typically, the ssUV-Vis may be used to give insight into the dye loading on the surface, because the absorption spectra depend on the concentration of the immersing dye solution and the sensitization time. Since the exact amount of the dye adsorbed on the surface is not known, arbitrary units of absorbance are used, and spectra are compared with that of a standard dye, in this case N719.

**Fig. 6:**
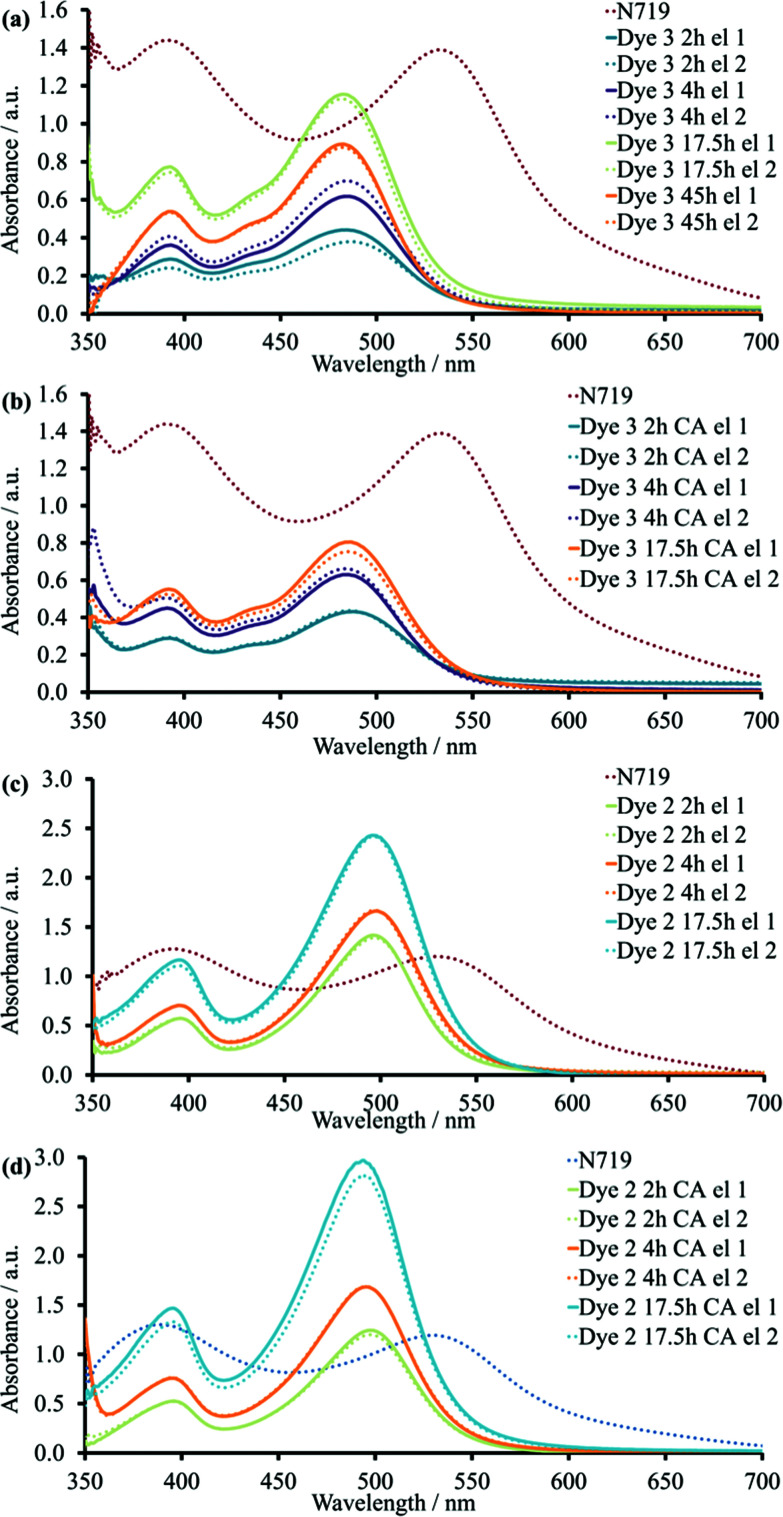
ssUV-Vis spectra of transparent TiO_2_ electrodes with adsorbed dye **2** or **3**: (a) Dye **3** sensitized in the absence of CA for different dipping times; (b) Dye **3** sensitized in the presence of CA for different dipping times; (c) Dye **2** sensitized in the absence of CA for different dipping times; (d) Dye **2** sensitized in the presence of CA for different dipping times. El – transparent TiO_2_ electrode.

For complex **3**, the band assigned to charge transfer to pyridine exhibited a blue shift from 510 to ≈482 nm (Fig. 6a). The bands with maxima at ≈434 and ≈393 nm were similar to those observed in MeCN. For complex **2**, the *λ*_max_ was also blue-shifted to *ca.* 497 nm compared to the UV-Vis spectrum in MeCN solution (Fig. 6c). However, the second MLCT band at higher energies was unchanged with λ_max_ ≈396 nm. Blue or red-shifting of the absorption maximum on going from solution to solid-state may indicate either H- or J-type aggregation, respectively.^22^

#### The influence of sensitization time and the presence of coadsorbent

The absorbance of complexes **2** and **3** increased with longer dipping times on going from 2 to 17.5 hours. This effect indicated changes in the dye load on the surface and was reflected in the enhanced *J*_SC_ values for both complexes with longer sensitization time. The ssUV-Vis spectra of TiO_2_/dye **2** exhibited higher absorption than TiO_2_/**3** for all three time periods (Fig. 6a and c). This observation could indicate a difference in the dye arrangement on the surface between homo- and heteroleptic complexes despite their similarities in general structure. It is important to note that **3** and **2** contain one and two CO_2_H anchoring groups, respectively.

As detailed above, our optimizations of dye/coadsorbent combinations for **1**^30^ had shown that the presence of CA reduced the dye load on the TiO_2_ surface. For complex **3**, the same tendency was found. The addition of CA decreased the difference in absorbance values at ≈482 nm for **3** between 17.5 and 4 hours (Fig. 6b), and this was in agreement with similar performance of DSCs with corresponding immersion times. For DSCs containing **3** and without CA, dipping times of 2 or 4 hours led to difference in the ssUV-Vis spectra (Fig. 6a). This observation illustrated that there was no competition between dye and CA for the duration of the dye-adsorption. Due to this, the enhanced PCE of DSCs dipped for 4 hours with CA in comparison to the corresponding set without CA corresponded to the suppressed recombination between surface and electrolyte. The increase in the immersion time from 17.5 to 45 hours resulted in a reduction of absorbance. Despite the logical assumption that longer dipping times should favour adsorption of more dye molecules on the surface, it could also bring the system into an equilibrium state between surface and solution. This would result in partial detachment of dye from the semiconductor.

In the case of complex **2**, the adsorption was enhanced on going from 2 to 17.5 hours (Fig. 6c). However, in contrast to complex **3**, the addition of CA to the dye-bath did not have an influence on intensity of ssUV-Vis absorption spectrum (Fig. 6d). The presence of two anchoring groups changes the ways of dye arrangement on the semiconductor surface and could result in stronger binding.

#### Thermogravimetric analysis

Due to the different loads of the homoleptic dye **2** and heteroleptic dye **3** on the surface that were indicated from differences in the ssUV-Vis spectra, thermogravimetric analysis (TGA) was performed in order to obtain a better understanding of the surface functionalization. An FTO/TiO_2_ electrode was sensitized by immersing the electrode into a dye bath for 17.5 hours in the absence of CA (0.05 mM dye-bath concentration). Afterwards, the TiO_2_ was scratched off the electrode, and the resulted powder was used for the TGA measurements.

**Fig. 7:**
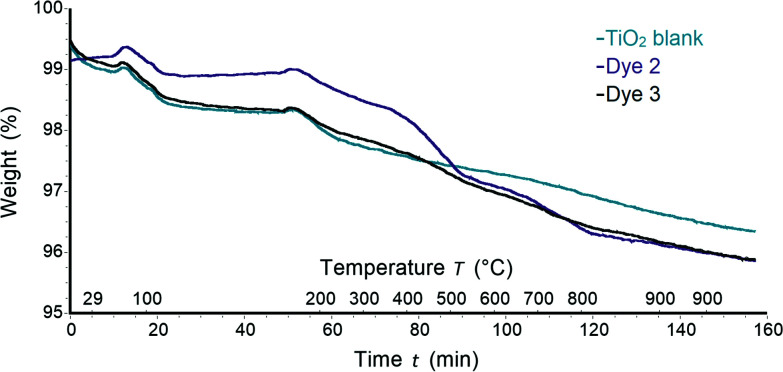
TGA curves for blank TiO_2_ (green), TiO_2_ sensitized with dye **2** (purple) and TiO_2_ sensitized with dye **3** (grey).

The weight losses of 0.32–0.59% and 0.22–0.35% (<120 °C and <220 °C, respectively) corresponded to loss of physisorbed and chemisorbed water^31^ and were observed for all samples including functionalized TiO_2_ and a control with no adsorbed dye (Fig. 7). Between 220 and 900 °C non-functionalized TiO_2_ had 1.6% weight lost (Fig. S3†). In the following discussion, only the weight losses corresponding to decomposition of dye molecules will be described, and the weight loss corresponding to TiO_2_ was subtracted from each TGA trace. The decomposition of each pristine dye was also analysed by TGA and the results were taken into account for mass loss calculations of sensitized TiO_2_ (Fig. S4†). A weight loss of 71.5% was detected for complex **3**. Mass peaks at *m*/*z* 41.0, 43.0 and 44.0 were assigned to acetonitrile, propylium and carbon dioxide arising from ligand decomposition (Fig. S4a†). Identical mass peaks were detected for complex **2** (Fig. S4b†). However, a higher weight loss of 78.2% was observed compared to **3**. Once the dye was deposited on TiO_2_, the loss of CO_2_ was detected with corresponding mass peaks at *m*/*z* 44.0 for all complexes (Fig. S5a and b†). It is important to note that no peaks at *m*/*z* 44.0 were detected for non-functionalized TiO_2_. Thus, the loss of CO_2_ could be attributed to decomposition and subsequent detachment of complexes from TiO_2_ surface. In the case of the homoleptic complex **2** with *n*-butyl chains a weight loss of 1.61% was observed. Dye **3** exhibited a similar weight loss of 0.77%. The variance in weight loss between samples with **2** and **3** deposited on TiO_2_ supports the proposal of different binding models and dye arrangement on the surface for homoleptic and heteroleptic complexes.

#### Electrochemical impedance study

**Fig. 8:**
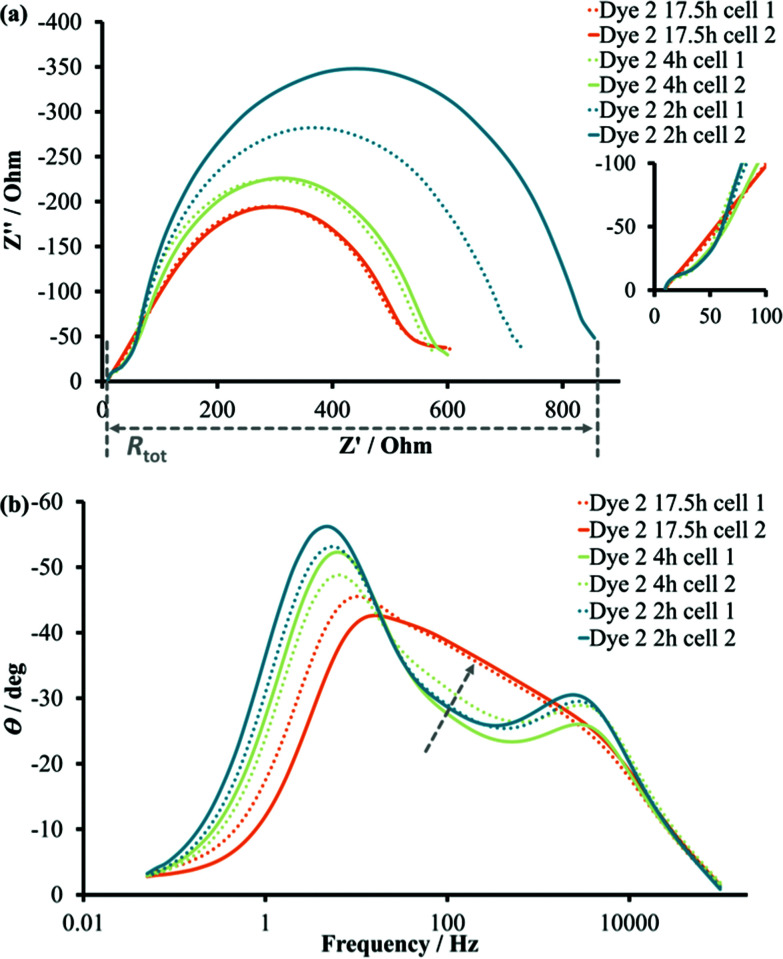
EIS plots for DSCs sensitized with 2 at different dipping times (2, 4 and 17.5 hours) in the absence of coadsorbent: (a) Nyquist plots, the expansion shows the high frequency region; (b) Bode plot, the arrow indicates the changes in the direction of Gerischer impedance.

Electrochemical impedance spectroscopy (EIS) can provide a deeper understanding of interfacial electronic processes in a DSC. Since it is a frequency dependent method, the different parts of the spectrum are attributed to electron processes happening on different timescales.^32^ For dye **2**, the experimental EIS curves were not fitted meaningfully. Nevertheless, the shapes of the curves can still provide important information about dominant recombination processes in DSCs. A strong dependency of total resistance (*R*_tot_, Fig. 8a) in the system on the sensitizing time was observed (the real axis *Z*′) and *R*_tot_ significantly decreased on going from 2 hours dipping time to 17.5 hours (in the absence of CA). The values of the imaginary axis *Z*′′ (capacitance) were also lower with longer dipping times. The shape of the Bode plot as well as the Nyquist plot changed in the direction of a Gerischer impedance^33^ for DSCs after 17.5 hours of sensitization (Fig. 8b). The area between high and low frequency peaks on the Bode plot indicated an increase in transport resistance for the longest dipping times. The decrease in *R*_tot_ was beneficial for electron injection and was in agreement with enhanced values of *J*_SC_ for dye **2** with longer time of sensitization. Yet, the Gerischer type of impedance for 17.5 h dipping indicated insufficient electron diffusion through the semiconductor and limited the PCE of these DSCs. In the case of a Gerischer impedance, the impedance shows a finite DC value despite the fact that the overall diffusion process is, in principle, semi-infinite with the assumption of an ‘infinitely large’ reservoir for the electrochemically inactive species.^34^ This interpretation allows an assumption that a higher dye-coverage on the surface increases the reaction rate between a dye-cation and *e*_inj_ during the diffusion process through the semiconductor. This results in the formation of electrochemically inactive species. As was shown in the ssUV-Vis spectra, longer dipping times resulted in an increased amount of dye **2** on the surface. Higher dye density offered more possibilities for the recombination between injected electron and oxidized dye. For dye **1** it was shown that injected electrons suffer from fast recombination with the oxidized Fe(ii) NHC complex.^16^ This would lead to lower electron density in the conduction band, a decrease in capacitance values, and an increase in the transport resistance. The addition of CA resulted in the same EIS trend for DSCs on going from shorter to longer dipping time.

For dye **3**, the fit was performed with a help of an electric circuit model (Fig. S6†). The parameters for fitted experimental curves are presented in Table 4 and the fitted curves are presented in Fig. S7.† As for DSCs with complex **2**, the sensitization time and the addition of a CA had a significant influence on the impedance of the DSC. The *R*_rec_ values decreased by a factor of two on going from 4 to 17.5 hours (without CA) of sensitization. The extended dipping time of 45 hours did not affect the recombination resistance. The values of chemical capacitance were increased from 4 to 17.5 hours, and this was consistent with the enhanced short circuit current density values. The addition of CA for the 4 hours-dipping DSCs set increased the *J*_SC_ values by more than 1 mA cm^−2^. This improvement corresponded to changes in *C*_μ_ from 528 to 845 μF and in *R*_tr_ from 10 to 3 Ω (Table 4 and Fig. S7c, d†). The recombination resistance decreased with the addition of CA (4 hours). The combination of respective factors indicated more efficient electron injection and fewer recombination processes taking place in the presence of CA. Interestingly, in the case of 17.5-hour sensitization the addition of CA decreased *C*_μ_ values from 905 to 665 μF (Fig. S7e, f and g, h†). This observation was associated with the competition between dye and CA on the surface and was supported by ssUV-Vis measurements. For all dipping times in the presence/absence of CA, the electron lifetime was significantly higher than transport time. Together with a diffusion length greater than the TiO_2_ thickness (*d* ≈14 μm), *τ* and *τ*_t_ offered an effective electron transport through the working electrode. One of the important observations was the simultaneous increase in electron lifetime together with chemical capacitance after 45 hours of sensitization. This was consistent with a higher electron density in the semiconductor compared with a 17.5 hours dipping time. However, the diffusion length remained similar (Table 4) meaning the higher dye load did not affect the recombination rate at the semiconductor/electrolyte interface. Series resistance and counter electrode parameters (*R*_Pt_ and *C*_Pt_) stayed constant and, expectedly, were not affected by changes at photoanode.

**Table 4:** Average EIS parameters for DSCs sensitized with **3**. Data for multiple DSCs are shown in Table S5†

Dye^a^	Time^b^/h	*R*_rec_/Ω	*C*_μ_/μF	*R*_tr_/Ω	*τ*/ms	*τ*_t_/ms	*L*_d_/μm	*R*_s_/Ω	*R*_Pt_/Ω	*C*_Pt_/μF
Dye 3	4	487	528	10	257	5	101	11	6	6
Dye 3 CA	4	355	845	3	297	2	155	11	5	6
Dye 3	17.5	257	905	3	231	3	144	12	5	6
Dye 3 CA	17.5	501	665	4	332	3	157	12	5	6
Dye 3	45	274	1555	3	429	4	149	12	7	6

a CA – in the presence of chenodeoxycholic acid (0.05 mM).

b The immersing time of working electrodes into the dye solution.

## Conclusions

Two new iron(ii) complexes with *n*-butyl side chains were employed in *n*-type DSCs. Initial studies showed that cells with the homoleptic complex **2** had significantly lower PCE (0.05%) compared to those with the homoleptic dye **1** (0.66%). Such a low performance was a result of lower values of *J*_SC_ and *V*_OC_ together with a low ff. However, the optimization of the dye-bath concentration and the immersion time enhanced all parameters extracted from *J–V* curves. This improvement resulted in six times higher PCE of 0.29%. Interestingly, when the dye-bath concentration was decreased from 0.50 to 0.05 mM, the overall PCE was little affected (0.27–0.29%). The addition of CA did not have a significant influence on DSCs performance. Moreover, the EIS response was more influenced by changes in dipping times, but not by the presence of CA. Gerischer impedance was observed for 17.5-hour immersion, and a decrease in *R*_tot_ and *R*_tr_ was observed for shorter dipping times.

DSCs with the new heteroleptic dye **3** significantly improved their overall photoconversion efficiency compared to dyes **1** and **2**. The highest PCE of 0.94% (average of a range 0.93–0.95%) was observed for DSCs sensitized over 17.5 hours without addition of coadsorbents. The shorter immersion of 4 hours decreased PCE (0.46%) due to the reduction of both, *J*_SC_ and *V*_OC_ values. However, a further increase of dipping time to 45 hours also resulted in lower PCE of 0.67% in comparison to 17.5 hours. The presence of CA led to similar DSC performances of 0.71 and 0.74% for 17.5 and 4 hours immersions, respectively. Interestingly, in the case of 17.5 hours, the presence of CA decreased *J*_SC_ values, but in the case of 4 hours, *J*_SC_ values were enhanced in the presence of CA. These observations could be rationalized using impedance spectra. A decrease in chemical capacitance was observed with addition of CA for 17.5-hour dipping time, but in contrast, after 4 hours the presence of CA led to the increase of *C*_μ_. In the case of 45-hour immersion, *C*_μ_ values had a remarkable increase in combination with the electron lifetime. However, the diffusion length stayed unchanged. Thus, the recombination rate at semiconductor/electrolyte interface was not affected despite the changes on the dye load on the surface.

The differences in DSC performance were strongly affected by the molecular structure of the dye. The variation in dye loading on the surface was supported by ssUV-Vis measurements in the presence/absence of CA. For complex **2**, absorbance maxima were not affected by addition of CA. On the other hand, a decrease in absorption intensity was observed for dye **3** in the presence of CA. Moreover, different weight losses of TiO_2_ sensitized with **2** or **3** were observed during thermogravimetric analysis. These observations lead us to propose different binding models on the surface for homoleptic and heteroleptic complexes.

## Author contributions

Conceptualization – M. B.; investigation – M. B. and V. W.; project administration, funding acquisition and supervision – C. E. H and E. C. C.; writing – original draft – M. B.; writing – review and editing – C. E. H. and E. C. C.

## Conflicts of interest

There are no conflicts to declare.

## Supplementary Material

DT-050-D1DT03252F-s001
