# Supplementary material for: The influence of alkyl chains on the performance of DSCs employing iron(ii) N-heterocyclic carbene sensitizers
Source: Dalton Trans. 2021 Oct 20;50(46):16961–9. doi: 10.1039/d1dt03252f (PMC8631001; doi:10.1039/d1dt03252f)
Supplement: DT-050-D1DT03252F-s001 [file DT-050-D1DT03252F-s001.pdf]

Electronic Supplementary Material (ESI) for Dalton Transactions.
This journal is © The Royal Society of Chemistry 2021

Supporting Information to accompany:

The influence of alkyl chains on the performance of DSCs employing iron(II) *N*-heterocyclic carbene sensitizers

Mariia Becker, Vanessa Wyss, Catherine E. Housecroft and Edwin C. Constable

Department of Chemistry, University of Basel, BPR 1096, Mattenstrasse 24a, 4058 Basel, Switzerland

Contents

General considerations for measurements 3
Synthesis and characterization of complexes 2 and 3 3
Synthesis of complex 2 3
Synthesis of complex 3 5
DSC characterization 7
Parameters extracted from *J-V* curves for multiple DSCs 7
TGA curves 8
Electric circuit model used for fitting EIS experimental data 9
EIS parameters for multiple DSCs 9
EIS plots for multiple DSCs 10
References 11

General considerations for measurements

Current density-voltage (*J–V*) measurements were made by irradiating from the photoanode side with a LOT Quantum Design LS0811 instrument (100 mW cm^{-2} = 1 sun at AM 1.5) and the simulated light power was calibrated with a silicon reference cell.

UV-Vis spectra were recorded on Shimadzu UV-2600 spectrophotometer. Solid-state UV-Vis spectra were measured on a VARIAN Cary-5000 spectrophotometer. Transparent TiO_2 electrodes were used as a reference for the solid-state absorption spectroscopic measurements.

For the EIS measurements, a ModuLab® XM PhotoEchem photoelectrochemical measurement system from Solartron Analytical was used. The impedance was measured at the open-circuit potential of the cell at a light intensity of 22 mW cm^{-2} (590 nm) in the frequency range 0.05 Hz to 100 kHz using an amplitude of 10 mV. The impedance data were analysed and fitted using ZView® sofware from Scribner Associates Inc.

N719 dye was purchased from Solaronix. Commercial working electrodes (opaque), platinum counter electrodes (Test Cell Platinum Electrodes Drilled) hot-melt sealing foil (Test Cell Gaskets, made from Meltonix 1170-60 sealing film, 60 microns thick) were obtained from Solaronix as well as. The conducting silver paint (colloidal suspension, 0.5 troy oz.) was obtained from SPI. HPLC grade solvents were used for solar cell fabrication, and were purchased from HPLC VWR and J.T. Baker.

Thermogravimetric analysis (TGA) was performed on a TGA5500 instrument (TA Instruments) coupled to a Discovery II MS, Cirrus 3, Mass Spectrometer, DMS. The analysis was carried under nitrogen, using a Barchart scanning method in the mass range 10-125. In all the experiments, the temperature of the TGA instrument was initially stabilized at 30°C for 10 min followed by heating at a rate of 10°C/min to 120°C. This temperature was maintained for 30 min. Afterwards each sample was heated at a rate of 10°C/min to 900°C. After 30 min at 900°C a sample was cooled down to ambient temperature.

Synthesis and characterization of complexes 2 and 3

Starting materials for synthesis were obtained in reagent grade from Avocado Research Chemicals Ltd, Sigma-Aldrich, Fluorochem, Alfa-Aesar, TCI, Carl Roth and Acros Organics. Dry solvents (crown cap or AcroSeal®) were purchased from Acros Organics. NMR solvents were obtained from Cambridge Isotope Laboratories Inc. and Apollo. Fluka silica gel 60 was used for flash chromatography. Thin layer chromatography (TLC) was performed with aluminium sheets covered with silica gel 60 (Merck).

^{1}H, $^{13}C\{^{1}H\}$ and $^{19}F\{^{1}H\}$ NMR spectra were recorded on Bruker Avance III-500 NMR spectrometers; spectra were recorded at 295 K. ^{1}H and ^{13}C spectra were referenced with respect to δ(TMS) = 0 ppm. High resolution mass spectrometry (HRMS) was performed on Bruker maXis 4G instrument. FTIR spectra were recorded on a Perkin Elmer UATR Two spectrophotometer.

Synthesis of complex 2

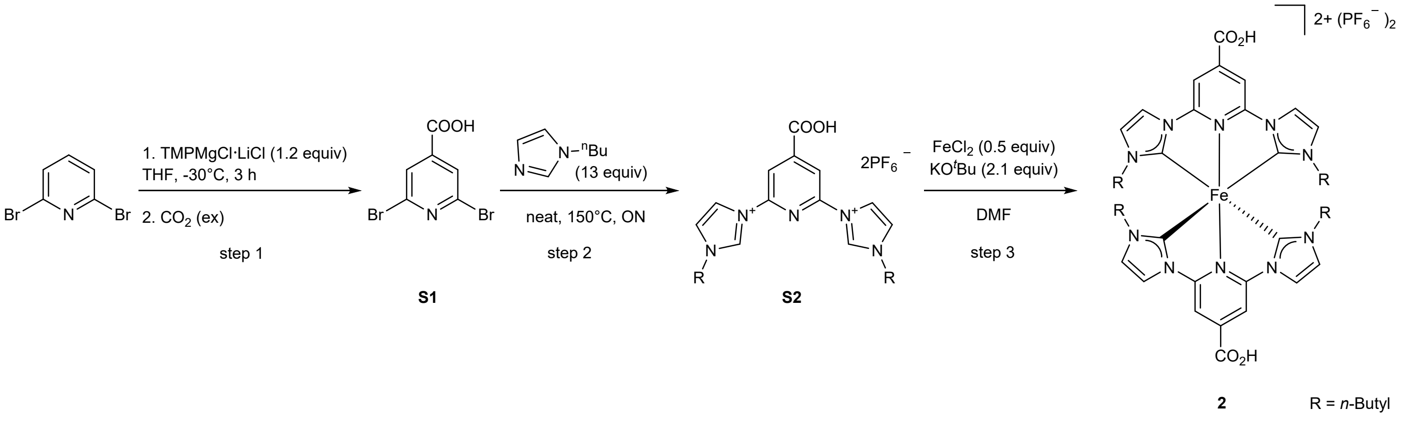

Fig. S 1. Synthetic route to afford target compound **2**.

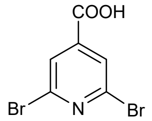

S1. The acid was synthesized according to the literature procedure used as reference.[1]

A flame-dried flask was charged with 2,6-dibromopyridine (1.0 equiv, 5.00 mmol, 1.18 g) under nitrogen. The flask was evaporated and refilled with nitrogen. Anhydrous THF (50 mL) was added and the solution was cooled to -30°C. TMPMgCl·LiCl (1.2 equiv, 6.00 mmol, 6.0 mL) was added dropwise for 15 min and the reaction mixture was stirred at the same temperature for 3 h. Afterwards the reaction was quenched with dry ice until gas evaluation stopped and the mixture was allowed to warm to room temperature overnight. The pH was adjusted (pH≈12) with 1M NaOH solution and the mixture was extracted with AcOEt (3 x 15 mL). The aq. layer was acidified with 1M H_2SO_4 to pH = 2 and extracted with AcOEt (3 x 15 mL). The combined organic fractions were dried over $MgSO_4$ and the solvent was removed under reduced pressure to obtain 2,6-dibromoisonicotinic acid as a white solid (1.65 mmol, 462 mg, 33%).

The NMR spectrum was in agreement with the literature.[2]

^{1}H-NMR (500 MHz, DMSO-d$_6$) *δ* / ppm: 14.24 (br. s, 1H), 7.97 (s, 2H).

^{13}C{^{1}H}-NMR (126 MHz, DMSO-d$_6$) *δ* / ppm: 163.5, 143.6, 140.8, 126.7.

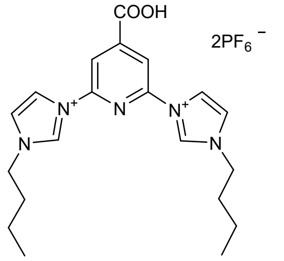

S2. The salt was synthesized according to a literature procedure.[3]

A pressure vial was charged with 2,6-dibromoisonicotinic acid (**S1**, 1.0 equiv, 0.37 mmol, 100 mg) and 1-butylimidazole (13 equiv, 4.59 mmol, 0.6 mL). The vial was sealed and the reaction mixture was stirred overnight at 150°C. Once the reaction had cooled down to room temperature, EtOH (5.0 mL), water (5.0 mL) was added. Then NH_4PF_6 sat. aq. solution (10 mL) was added. The mixture was acidified with 1M HCl aq. solution to pH = 2. Afterwards, EtOH (10 mL) was added and the precipitate was collected *via* filtration, washed with EtOH (3 x 10 mL) and dried under high vacuum at 70°C for 16 h to obtain 2,6-bis(3-butylimidazolium-1-yl)pyridine-4-carboxylate hexafluorophosphate as white solid (0.15 mmol, 100 mg, 43%).

MP: 156.7°C

IR (solid) / cm^{-1}: 1695 (w), 1545 (m), 1538 (m), 1449 (m), 1220 (m), 1143 (m), 1133 (m), 1110 (w), 1102 (w), 1086 (m), 1032 (m), 1015 (m), 864 (m), 856 (m), 829 (s).

HRMS: *m/z* 368.21, (calc. 368.21 [M-2PF$_6$]$^{2+}$).

^{1}H-NMR (500 MHz, DMSO-d$_6$) *δ* / ppm: 9.89 (t, *J* = 1.6 Hz, 2H), 8.37 (t, *J* = 1.9 Hz, 2H), 8.07 (s, 2H), 7.61 (t, *J* = 1.8 Hz, 2H), 3.77 (t, *J* = 7.2 Hz, 4H), 1.36 (dq, *J* = 9.6, 7.3 Hz, 4H), 0.86 – 0.76 (m, 4H), 0.40 (t, *J* = 7.4 Hz, 6H).

^{13}C{^{1}H}-NMR (126 MHz, DMSO- d$_6$) *δ* / ppm: 164.0, 146.1, 136.2, 123.7, 119.6, 113.8, 49.6, 31.0, 18.8, 13.3.

^{19}F{^{1}H}-NMR (471 MHz, DMSO-d$_6$) *δ* / ppm: -70.17 (d, *J* = 711.3 Hz).

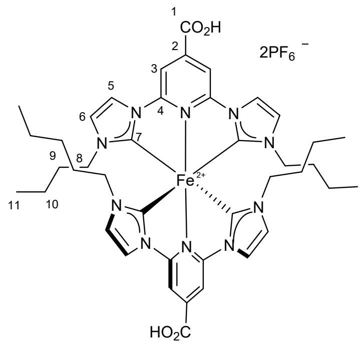

Complex 2. The iron(II) NHC complex **2** was synthesized according to an adapted literature procedure.[3]

A flask was charged with the ligand **S2** (2.0 equiv, 0.11 mmol, 60.0 mg) and KO^{t}Bu (7.0 eqiuv, 0.78 mmol, 87.2 mg) under inert conditions. Anhydrous $FeCl_2$ (1.0 equiv, 0.06 mmol, 14.1 mg) was dissolved in anhydrous DMF (3.0 mL), and the solution was transferred to solids. The reaction mixture was stirred at room temperature for 1 h. 1M H_2SO_4 aq. solution was added to adjust the pH to 2, then NH_4PF_6 (excess, 200 mg) was added, and the precipitate started to form. The precipitate was collected *via* filtration, washed with water (3 x 10 mL) and methyl tert-butyl ether (MTBE, 3 x 10 mL) to obtain bis(2,6-bis(3-butylimidazol-1-ylidene)pyridine-4-carboxylic acid)iron(II) hexafluorophosphate as a red solid (0.02 mmol, 16.0 mg, 33%).

IR (solid) / cm^{-1}: 3111 (w), 2961 (w), 2875 (w), 1724 (w), 1623 (w), 1579 (w), 1549 (w), 1482 (m), 1423 (w), 1367 (w), 1261 (m), 1237 (m), 1146 (m), 1122 (m), 1087 (m), 1017 (m), 976 (m), 834 (s).

HRMS: *m/z* 395.1677 (calc. 395.17 $[M-2PF_6]^{2+}$).

^{1}H-NMR (500 MHz, Acetone-d$_6$) δ / ppm: 8.86 (s, 1H, H^3), 8.64 (s, 1H, H^5), 7.55 (s, 1H, H^6), 2.91 (s, 2H, H^8), 0.98 (s, 2H, H^9), 0.79 (s, 3H, H^{10}), 0.69 (s, 4H, H^{11}).

^{13}C{^{1}H}-NMR (126 MHz, Acetone-d$_6$) δ/ppm: 198.5 (C^7), 164.8 (C^1), 155.6 (C^4), 141.3 (C^2), 127.2 (C^6), 118.7 (C^5), 105.9 (C^3), 49.8 (C^8), 33.9 (C^9), 20.3 (C^{10}), 13.9 (C^{11}).

^{19}F{^{1}H}-NMR (471 MHz, CD_3CN) δ / ppm: -72.96 (d, J = 706.4 Hz).

Synthesis of complex 3

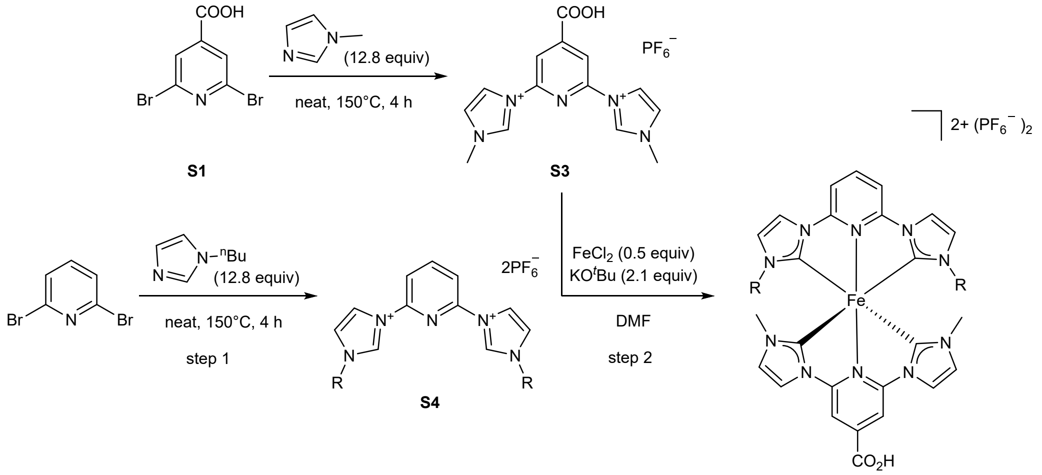

3 R = *n*-Butyl

Fig. S 2. Synthetic route to afford target compound **3**.

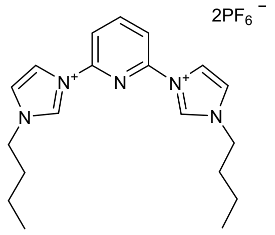

S3. The salt was synthesized according to a literature procedure.[3]

A pressure vial was charged with 2,6-dibromopyridine (1.0 equiv, 4.00 mmol, 948 mg) and 1-butylimidazole (13 equiv, 51.6 mmol, 6.4 mL). The vial was sealed and the reaction mixture was stirred overnight at 150°C. Once the reaction cooled down to room temperature, EtOH (5.0 mL), water (5.0 mL) was added. Then NH_4PF_6 sat. aq. solution (10 mL) was added. The mixture was acidified with 1M HCl aq. solution to pH = 2. Afterwards, EtOH (10 mL) was added and the precipitate was collected *via* filtration, washed with EtOH (3 x 10 mL) and dried under high vacuum at 70°C for 16 h to obtain 2,6-bis(3-butylimidazolium-1-yl)pyridine hexafluorophosphate as a white solid (0.30 mmol, 186 mg, 8%).

MP: 152.3°C

IR (solid) / cm^{-1}: 3160 (w), 2970 (w), 1615 (w), 1581 (w), 1533 (m), 1464 (m), 1416 (w), 1375 (w), 1331 (w), 1304 (w), 1220 (m), 1110 (m), 1081 (m), 1005 (w), 820 (s), 806 (s).

HRMS: *m/z* 162.61, (calc. 162.61 $[M-2PF_6]^{2+}$)., *m/z* 470.19 (calc. 470.19 ($-PF_6^-$)).

^{1}H-NMR (500 MHz, DMSO-d$_6$) δ / ppm: 10.27 (d, J = 1.8 Hz, 1H), 8.79 (t, J = 1.9 Hz, 1H), 8.61 (t, J = 8.1 Hz, 1H), 8.20 (d, J = 8.1 Hz, 1H), 8.17 (t, J = 1.9 Hz, 1H), 4.31 (t, J = 7.2 Hz, 2H), 2.04 – 1.79 (m, 2H), 1.42 – 1.31 (m, 2H), 0.96 (t, J = 7.4 Hz, 3H).

^{13}C{^{1}H}-NMR (126 MHz, DMSO-d$_6$) δ /ppm: 145.7, 145.1, 136.1, 124.2, 119.9, 114.7, 50.1, 31.5, 19.3, 13.8.

^{19}F{^{1}H}-NMR (471 MHz, CD_3CN) δ / ppm: -70.16 (d, J = 711.3 Hz).

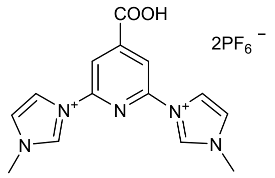

S4. The salt was synthesized according to a literature procedure.[3]

A pressure vial was charged with 2,6-dibromoisonicotinic acid (1.0 equiv, 0.98 mmol, 276 mg) and 1-methylimidazole (12.8 equiv, 12.5 mmol, 1 mL). The vial was sealed and the reaction mixture was stirred for 4 h at 150°C. Once the reaction cooled down to rt, water (5 mL) was added and NH_4PF_6 sat. aq. solution (10 mL) was added. The mixture was acidified with 1M H_2SO_4 to pH = 2. The precipitate was collected *via* filtration, washed with water (3 x 10 mL) and MTBE (3 x 10 mL) and dried under high vacuum at 70°C for 16 h to obtain 2,6-bis(3-methylimidazolium-1-yl)pyridine-4-carboxylate hexafluorophosphate as beige solid (0.44 mmol, 254 mg, 45%).

^{1}H-NMR (500 MHz, DMSO-d$_6$) δ / ppm: 10.41 (d, J = 1.6 Hz, 2H), 8.85 (t, J = 1.9 Hz, 2H), 8.53 (s, 2H), 8.04 (t, J = 1.8 Hz, 2H), 4.01 (s, 6H).

^{13}C{^{1}H}-NMR (126 MHz, DMSO-d$_6$) δ / ppm: 163.8, 145.6, 136.6, 124.8, 119.3, 113.5, 36.5.

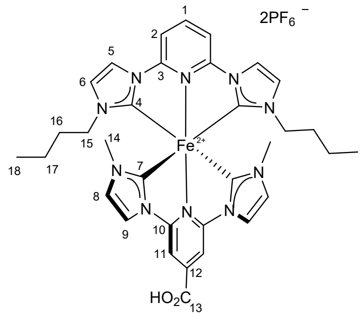

Complex 3. The iron(II) NHC complex **3** was synthesized according to an adapted literature procedure. [4]

A flask was charged with anhydrous $FeCl_2$ (1.0 equiv, 0.25 mmol, 31.7 mg) and the ligand **S3** (2.0 equiv, 0.50 mmol, 288 mg) under nitrogen. Anhydrous DMF (1.5 mL) was added followed by KO^{t}Bu (1M solution in THF, 3.2 eqiuv, 0.80 mmol, 0.8 mL). The reaction mixture was stirred at room temperature overnight. 1M H_2SO_4 was added to adjust the pH to 2, then HPF_6 (3% aq. solution, 10 mL) was added until the precipitate started to form. The mixture was kept at 4°C overnight. Then the precipitate was collected *via* filtration, washed with water (3 x 10 mL) and MTBE (3 x 10 mL). Further, the red solid was purified by flash chromatography on silica gel with acetone/water/KNO_3 sat. aq. solution (10:3:0.5) as eluent. The fraction with the target compound was collected, the solvent was evaporated and the residue was re-dissolved in water at 60°C. Then the homogeneous solution was allowed to cool down to room temperature. HPF_6 (6% aq. solution) was added until the precipitate started to form. The reaction mixture was stirred at rt for 1 h. Afterwards, the red precipitate was collected, washed with water (3 x 10 mL) and MTBE (3 x 10 mL) and dried to obtain (2,6-(3-butylimidazol-1-ylidene)pyridine)(2,6-bis(3-methylimidazol-1-ylidene)pyridine-4-carboxylic acid)iron(II) hexafluorophosphate as a dark red solid (0.01 mmol, 12.0 mg, 5%).

IR: 3177 (w), 3142 (w), 2966 (w), 2932 (w), 2877 (w), 1699 (m), 1627 (m), 1543 (w), 1494 (m), 1480 (s), 1428 (m), 1344 (w), 1269 (s), 1237 (m), 1105 (w), 1087 (m), 1018 (m), 1001 (m), 949 (w), 825 (s), 784 (s), 732 (m), 686 (s), 553 (s), 487 (s).

HRMS: m/z 331.1262 (calc. 331.1259 $[M{-}2PF_6]^{2+}$).

^{1}H-NMR (500 MHz, CD_3CN) δ / ppm: 8.33 (s, 2H^{11}), 8.25 (t, J = 8.2 Hz, 1H^1), 8.19 (d, J = 2.2 Hz, 2H^9), 8.04 (d, J = 2.2 Hz, 2H^5), 7.76 (d, J = 8.2 Hz, 2H^2), 7.06 (dd, J = 2.2, 1.5 Hz, 4H^{6,8}), 2.58 – 2.51 (m, 12H^{14,15}), 0.87 – 0.77 (m, 4H^{16}), 0.70 – 0.62 (m, 10H^{17,18}).

^{13}C{^{1}H}-NMR (126 MHz, CD_3CN) δ / ppm: 200.5 (C^7), 198.5 (C^4), 165.1 (C^{13}), 154.4 (C^3), 140.4 (C^1), 128.1 (C^8), 125.9 (C^6), 117.8 (C^5), 117.5 (C^9), 106.4 (C^2), 105.3 (C^{11}), 49.5 (C^{15}), 35.3 (C^{14}), 33.5 (C^{16}), 20.2 (C^{17}), 13.6 (C^{18}). (Tertiary carbon peaks C^{10} and C^{11} were not observed)

^{19}F{^{1}H}-NMR (471 MHz, CD_3CN) δ / ppm: -72.95 (d, J = 706.3 Hz).

DSC characterization

Parameters extracted from *J-V* curves for multiple DSCs

Table S 1. Parameters for four DSCs sensitized with N719 (0.3 mM in EtOH) with corresponding average values.

Dye	Time / h	J_{SC} / mA cm^{-2}	V_{OC} / mV	*ff* / %	PCE / %
N719	17.5	14.56	629	65	5.95
N719	17.5	15.34	636	66	6.48
N719	17.5	14.50	667	65	6.33
N719	17.5	14.41	672	69	6.68
Average value	–	14.70	651	66	6.36

Table S 2. Parameters for fully masked DSCs sensitized with dye **2** in the presence of chenodeoxycholic acid. Dye concentration was 0.50 mM, chenodeoxycholic acid concentration was 0.10 mM.

Dye[a]	Time / h	J_{SC} / mA cm^{-2}	V_{OC} / mV	*ff* / %	PCE / %	Rel. PCE / %[b]
Dye 2 CA cell 1	2	1.30	220	62	0.18	2.8
Dye 2 CA cell 2	2	1.35	231	62	0.19	3.0
Dye 2 CA cell 1	4	1.21	252	62	0.19	3.0
Dye 2 CA cell 2	4	1.41	259	65	0.24	3.8
Dye 2 CA cell 1	17.5	0.63	143	52	0.05	0.8
Dye 2 CA cell 2	17.5	0.63	122	46	0.04	0.6
Dye 2 CA cell 3	17.5	0.50	113	47	0.03	0.5
Dye 2 CA cell 4	17.5	0.88	133	51	0.06	0.9
Dye 2 cell 1	17.5	0.65	105	45	0.03	0.5
Dye 2 cell 2	17.5	0.62	109	44	0.03	0.5
Dye 2 cell 3	17.5	0.57	112	45	0.03	0.5
Dye 2 cell 4	17.5	0.46	131	48	0.03	0.5

[a] CA – chenodeoxycholic acid; [b] Relative efficiencies are given with respect to N719 PCE set as 100%.

Table S 3. Parameters for fully masked DSCs sensitized with dye **2** in the presence of chenodeoxycholic acid. Dye concentration was 0.05 mM, chenodeoxycholic acid concentration was 0.05 mM.

Dye[a]	Time / h	J_{SC} / mA cm^{-2}	V_{OC} / mV	*ff* / %	PCE / %	Rel. PCE / %[b]
Dye 2 cell 1	2	1.41	325	64	0.29	4.6
Dye 2 cell 2	2	1.42	331	63	0.29	4.6
Dye 2 cell 3	2	1.38	339	63	0.29	4.6
Dye 2 cell 4	2	1.41	348	61	0.30	4.7
Dye 2 CA cell 1	2	1.45	333	63	0.30	4.7
Dye 2 CA cell 2	2	1.39	334	63	0.29	4.6
Dye 2 CA cell 3	2	1.53	303	63	0.29	4.6
Dye 2 cell 1	4	1.58	296	63	0.29	4.6
Dye 2 cell 2	4	1.57	291	63	0.29	4.6
Dye 2 cell 3	4	1.52	315	63	0.30	4.7
Dye 2 cell 4	4	1.54	301	62	0.29	4.6
Dye 2 CA cell 1	4	1.53	283	63	0.28	4.4
Dye 2 CA cell 2	4	1.55	283	63	0.28	4.4
Dye 2 CA cell 3	4	1.61	264	63	0.27	4.2
Dye 2 cell 1	17.5	1.58	275	64	0.28	4.4
Dye 2 cell 2	17.5	1.59	283	64	0.29	4.6
Dye 2 cell 3	17.5	1.65	263	63	0.27	4.2
Dye 2 cell 4	17.5	1.73	260	63	0.28	4.4
Dye 2 CA cell 1	17.5	1.49	262	62	0.24	3.8
Dye 2 CA cell 2	17.5	1.61	262	63	0.26	4.1
Dye 2 CA cell 3	17.5	1.60	252	62	0.25	3.9

[a] CA – chenodeoxycholic acid.

Table S 4. Parameters for fully masked DSCs sensitized with dye **3** in the presence of chenodeoxycholic acid. Dye concentration was 0.05 mM, chenodeoxycholic acid concentration was 0.05 mM.

Dye[a]	Time / h	J_{SC} / mA cm^{-2}	V_{OC} / mV	*ff* / %	PCE / %	Rel. PCE / %[b]
Dye 3 cell 1	4	1.99	364	60	0.44	6.9
Dye 3 cell 2	4	2.12	381	62	0.50	7.9
Dye 3 cell 3	4	2.14	376	61	0.49	7.7
Dye 3 cell 4	4	1.97	360	61	0.43	6.8
Dye 3 CA cell 1	4	2.67	394	57	0.60	9.4
Dye 3 CA cell 2	4	3.29	391	59	0.75	11.8
Dye 3 CA cell 3	4	3.41	402	59	0.81	12.7
Dye 3 CA cell 4	4	3.49	396	58	0.80	12.6
Dye 3 cell 1	17.5	3.52	442	61	0.95	14.9
Dye 3 cell 2	17.5	3.78	408	61	0.95	14.9
Dye 3 cell 3	17.5	3.73	400	62	0.93	14.6
Dye 3 CA cell 1	17.5	3.12	390	60	0.73	11.5
Dye 3 CA cell 2	17.5	2.93	399	60	0.70	11.0
Dye 3 CA cell 3	17.5	2.94	403	59	0.70	11.0
Dye 3 cell 1	45	3.43	356	56	0.68	10.7
Dye 3 cell 2	45	3.24	349	55	0.62	9.7
Dye 3 cell 3	45	3.45	366	56	0.70	11.0
Dye 3 cell 4	45	3.40	359	55	0.67	10.5

[a] CA – chenodeoxycholic acid.

TGA curves

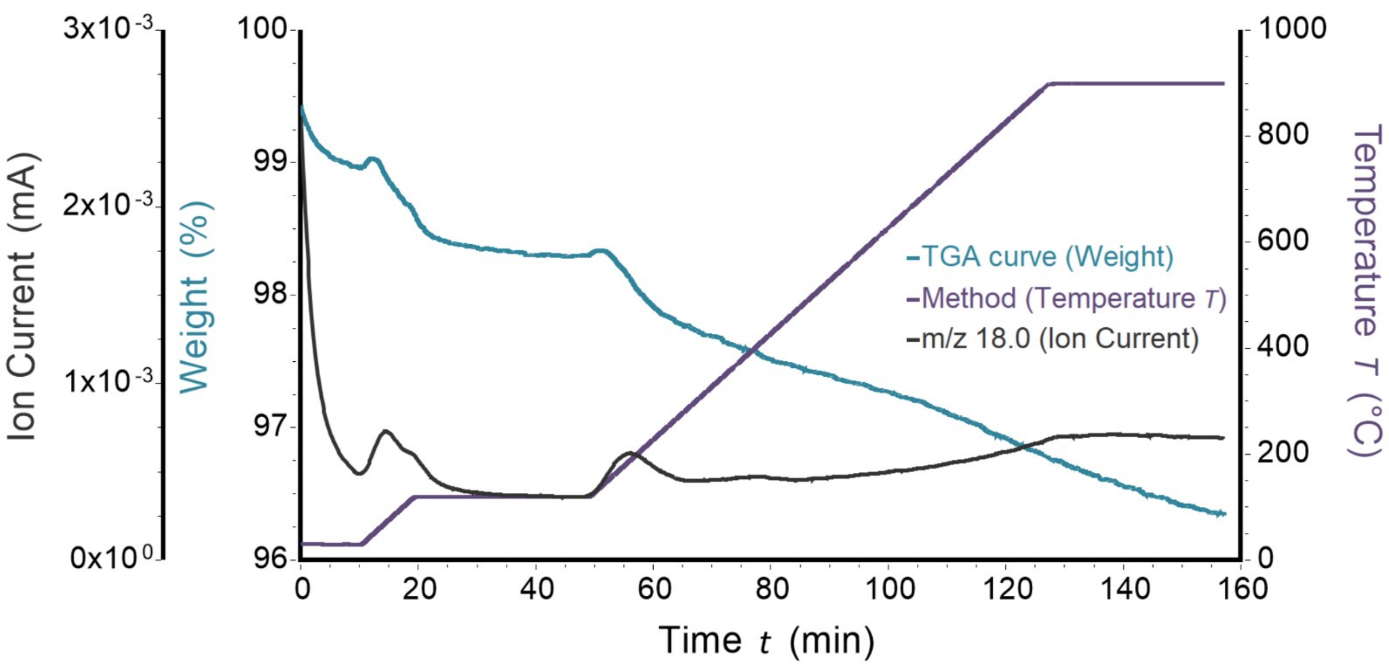

Fig. S 3. TGA and mass spectrometric traces for blank TiO_2.

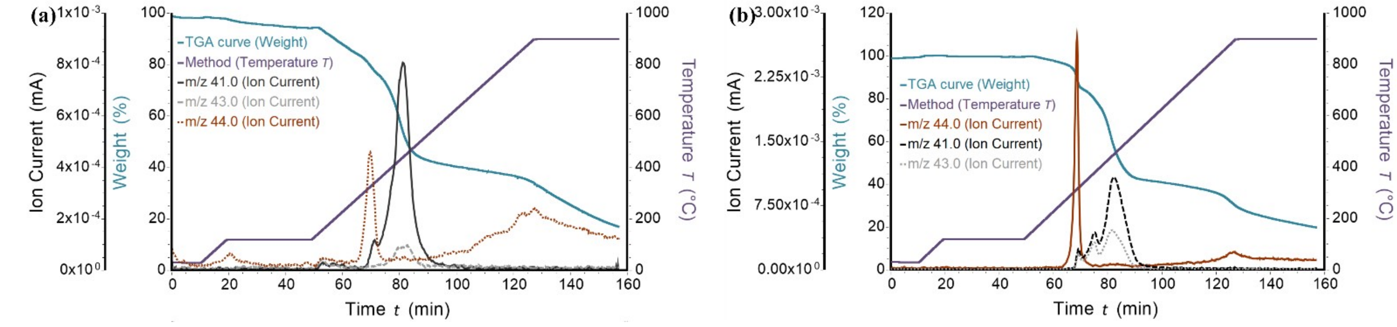

Fig. S 4. TGA and mass spectrometric traces for (a) dye **3**; (b) dye **2**.

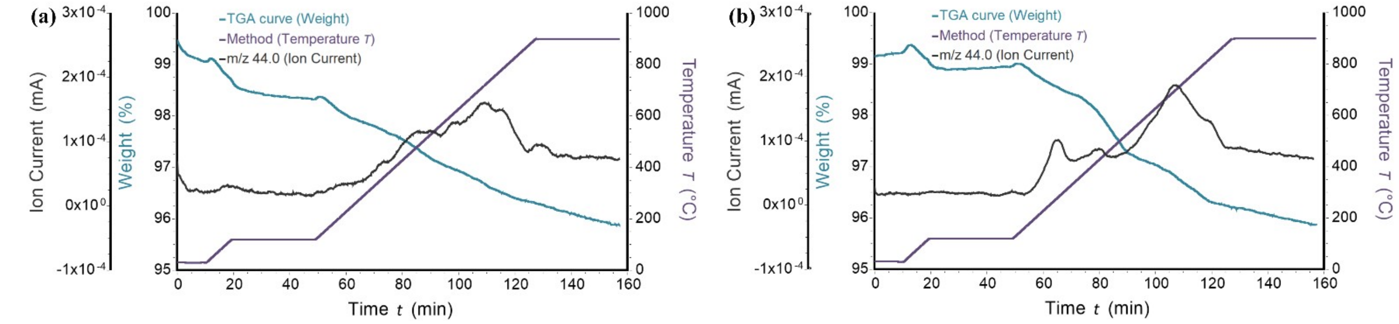

Fig. S 5. TGA and mass spectrometric traces for TiO_2 sensitized with (a) dye **3**; (b) dye **2**.

Electric circuit model used for fitting EIS experimental data

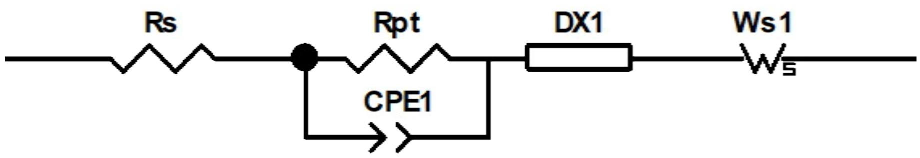

Fig. S 6. The circuit model consists of five elements and includes a series resistance (Rs), a resistance (R_{Pt}) and a constant phase element (CPE_{Pt}) to model a counter electrode, an extended distributed element (DX1) to represent the mesoporous TiO_2/electrolyte interface as a transmission line model, and a Warburg element (Ws) to represent the diffusion of the electrolyte.

EIS parameters for multiple DSCs

Table S 5. EIS parameters for DSCs sensitized with dye **3** at different immersion times.

Dye[a]	Time / h	R_{rec} / Ω	C_{μ} / μF	R_{tr} / Ω	τ / ms	τ_t / ms	L_d / μm	R_s / Ω	R_{Pt} / Ω	C_{Pt} / μF
Dye 3 cell 1	4	493	499	12	246	6	91	12	7	6
Dye 3 cell 2	4	494	607	8	300	5	112	11	6	6
Dye 3 cell 3	4	457	560	7	256	4	116	10	5	7
Dye 3 cell 4	4	506	448	13	227	6	87	12	6	6
Dye 3 CA cell 1	4	501	774	3	388	2	191	10	4	6
Dye 3 CA cell 2	4	273	831	3	227	2	134	10	5	6
Dye 3 CA cell 3	4	316	858	3	271	3	144	11	5	6
Dye 3 CA cell 4	4	331	916	3	303	3	151	10	5	6
Dye 3 cell 1	17.5	290	925	6	268	5	99	12	6	6
Dye 3 cell 2	17.5	211	1020	1	215	1	171	13	5	6
Dye 3 cell 3	17.5	271	771	2	209	2	163	12	4	7
Dye 3 CA cell 1	17.5	422	668	5	282	4	125	13	5	6
Dye 3 CA cell 2	17.5	535	666	2	356	2	206	12	5	6
Dye 3 CA cell 3	17.5	545	660	5	359	4	141	12	6	6
Dye 3 cell 1	45	237	1492	3	353	4	124	12	8	6
Dye 3 cell 2	45	274	1584	2	434	3	164	12	7	6
Dye 3 cell 3	45	264	1452	2	384	3	161	13	8	6
Dye 3 cell 4	45	322	1691	3	544	5	145	12	7	6

[a] CA – chenodeoxycholic acid.

EIS plots for multiple DSCs

(a)

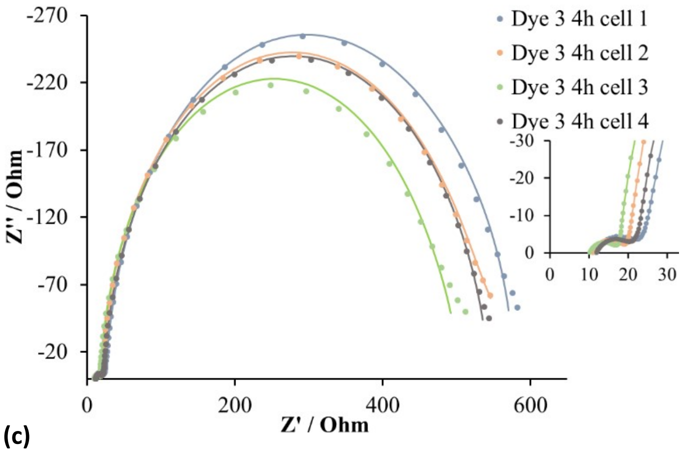

(b)

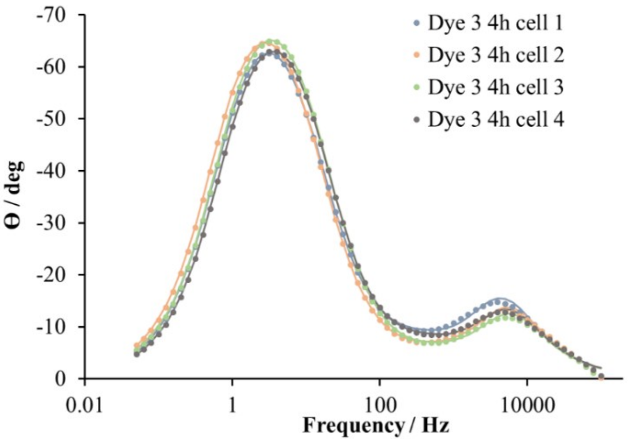

(c)

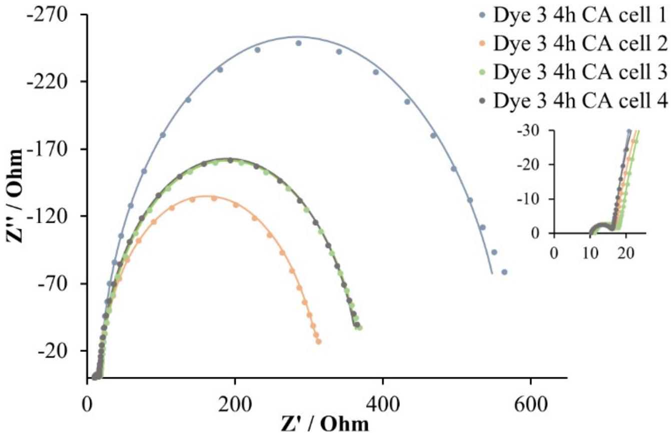

(d)

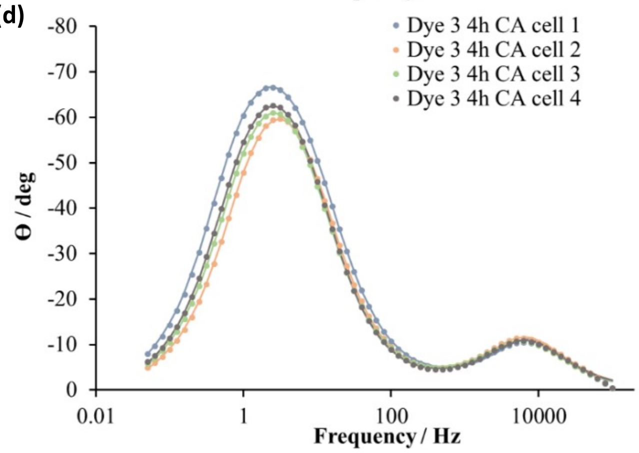

(f)

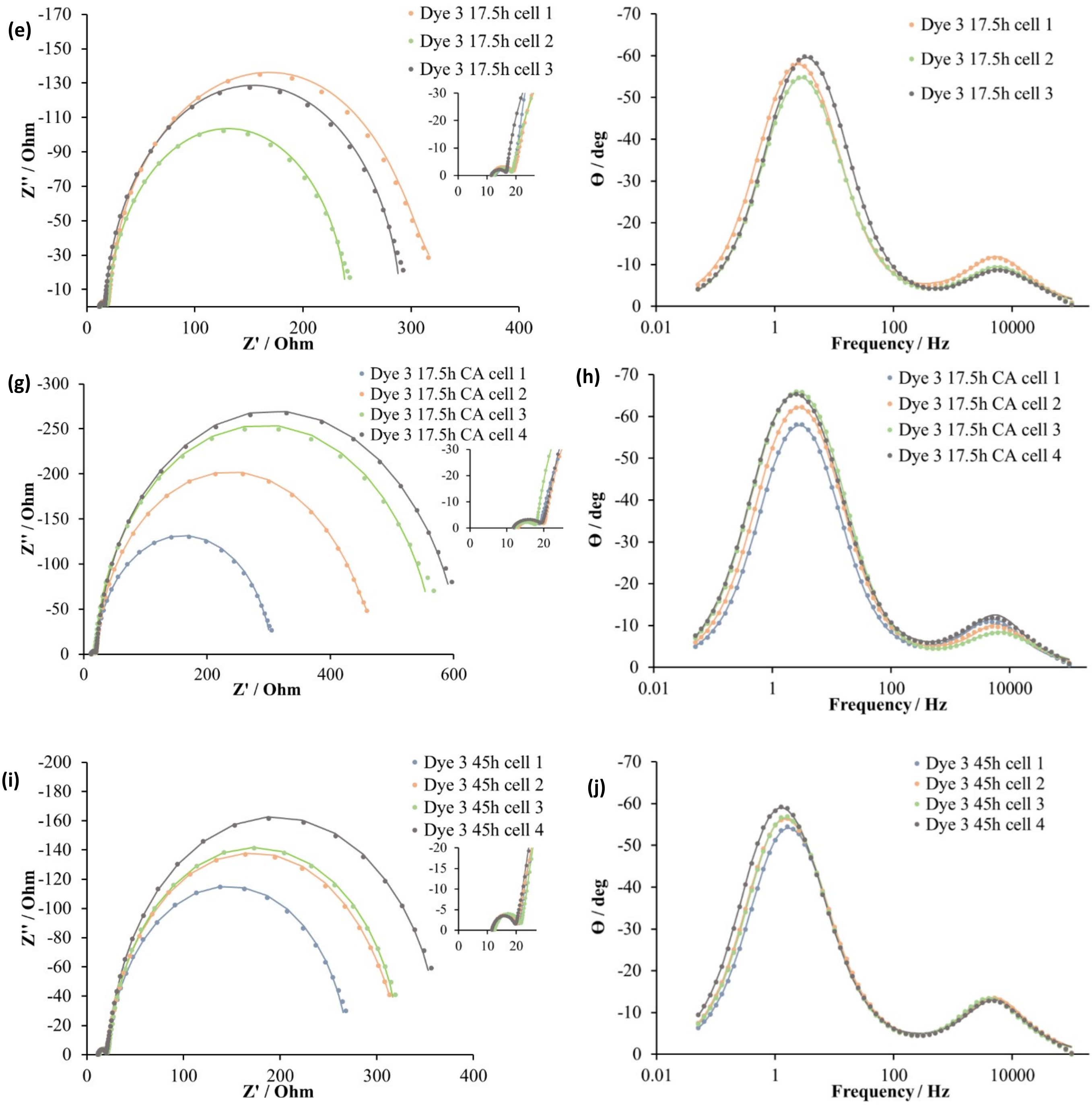

Fig. S 7. EIS plots for multiple DSCs sensitized with dye **3**. Solid lines correspond to fitted curves, dotted lines correspond to experimental data: (a) Nyquist plots for DSCs with 4 hours immersion, the expansion shows the high frequency region; (b) Bode plots for DSCs with 4 hours immersion; (c) Nyquist plots for DSCs with 4 hours immersion in the presence of CA, the expansion shows the high frequency region; (d) Bode plots for DSCs with 4 hours immersion in the presence of CA; (e) Nyquist plots for DSCs with17.5 hours immersion, the expansion shows the high frequency region; (f) Bode plots for DSCs with 17.5 hours immersion; (g) Nyquist plots for DSCs with 17.5 hours immersion in the presence of CA, the expansion shows the high frequency region; (h) Bode plots for DSCs with 17.5 hours immersion in the presence of CA; (i) Nyquist plots for DSCs with 45 hours immersion, the expansion shows the high frequency region; (j) Bode plots for DSCs with 45 hours immersion.

References

1. C. J. Rohbogner, S. H. Wunderlich, G. C. Clososki and P. Knochel, *Eur. J. Org. Chem.*, 2009, **11**, 1781-1795.
2. W. R. Bowman, M. O. Cloonan, A. J. Fletcher and T. Stein, *Org. Biomol. Chem.*, 2005, **3**, 1460-1467.

3. T. Duchanois, T. Etienne, C. Cebrián, L. Liu, A. Monari, M. Beley, X. Assfeld, S. Haacke and P. C. Gros, *Eur. J. Inorg. Chem.*, 2015, **14**, 2469.
4. A. Reddy Marri, E. Marchini, V. D. Cabanes, R. Argazzi, M. Pastore, S. Caramori and P. C. Gros, *J. Mater. Chem. A*, 2021, **9**, 3540-3554.
